# Advances in Feline Viruses and Viral Diseases

**DOI:** 10.3390/v13050923

**Published:** 2021-05-17

**Authors:** Julia A. Beatty, Katrin Hartmann

**Affiliations:** 1 Department of Veterinary Clinical Sciences and Centre for Companion Animal Health, Jockey Club College of Veterinary Medicine and Life Sciences, City University, Hong Kong, China; 2 Medizinische Kleintierklinik, Centre for Clinical Veterinary Medicine, LMU Munich, 80539 Munich, Germany; hartmann@lmu.de

## 1. Introduction

Viral diseases play a very important role in feline medicine, and research on feline viruses and viral diseases is a well-established field that helps to safeguard the health of domestic cats and non-domestic felids, many of which are endangered. This research also informs an expanding multibillion-dollar companion animal veterinary industry and, both directly and indirectly, contributes to the health of humans and the other animals that interact with cats. Last but not least, feline virus infections serve as important animal models for human viral diseases, such as immunodeficiency or coronavirus infections.

This Special Issue brings together 27 papers, including four reviews, covering diverse aspects of feline viruses and viral diseases. The recent emergence of the COVID-19 pandemic, caused by a highly pathogenic coronavirus of animal origin, SARS-CoV-2, further emphasizes the relevance of companion animal virology to global One Health.

### 1.1. Coronaviruses

Sadly, familiar to veterinarians, feline infectious peritonitis (FIP) is a fatal disease caused by feline coronavirus (FCoV) variants. In comprehensive review articles, Felten and Hartmann provide a timely update on the clinical diagnosis of FIP [1], while Jaimes et al. present a novel approach to coronavirus classification considering the genetic, structural, and functional characteristics of FCoV serotypes and S proteins, concluding that serotypes I and II FCoVs are distinct viruses [2].

Malbon and colleagues report that, in addition to primary immune organs, cardiac and hepatic tissues can represent a significant source of inflammatory cytokines, contributing to the pathogenesis of systemic inflammation characteristic of the FIP [3]. In a colony infected with the ubiquitous, usually subclinical, enteric FCoV, Pearson et al. identified strong systemic IgG and mucosal IgA responses but no virus-specific mucosal T-cell IFNγ responses or histologic abnormalities, suggesting that enteric FCoV is controlled by humoral mucosal and systemic responses [4].

In an eerily prophetic study, Zhao et al. demonstrate cross reactivity of feline sera against the S1 receptor-binding unit of coronavirus spike proteins from a range of species, noting the relevance for diagnostics as well as the potential for cats to be involved in cross-species coronavirus transmission [5].

### 1.2. Parvoviruses

Feline panleukopenia virus (FPV) is a re-emerging pathogen. The importance of routine vaccination for pet cats against FPV is highlighted in two reports of outbreaks of feline panleukopenia, a vaccine-preventable, fatal parvoviral disease. In the first report, Jenkins et al. report high FPV antibody prevalence in cats from outbreak and non-outbreak regions in Australia [6]. Less than half of the cats studied were known to be vaccinated, although 94% recorded protective titers, suggesting that cats are commonly exposed to *Carnivore protoparvovirus 1* in the field. The continued need to determine the level of vaccine coverage providing herd immunity against FPV is highlighted.

In the second report, phylogenetic analyses by van Brussel et al. identified distinct lineages of FPV from outbreaks in Australia, New Zealand and the United Arab Emirates [7]. Inadequate vaccination of shelter-housed cats was a common factor suggested to precipitate multiple re-emergence events.

### 1.3. Caliciviruses

Feline calicivirus (FCV), a frequent cause of upper respiratory tract disease, is increasingly associated with outbreaks of severe virulent systemic disease (VSD), but the basis for the increased pathogenicity of this clinical variant is elusive. In a fresh approach, Brunet et al. report, for the first time, consistent differences in capsid protein variable region amino acids between classical and virulent FCV strains using multiple correspondence analysis [8]. Barrera-Vasquez et al. report that survivin overexpression reduced FCV infection in vitro, correlating with reduced expression of the FCV receptor, the feline junctional adhesion molecule (fJAM1) [9].

Highly relevant to clinical practice, findings by Bergmann et al. on FCV antibody testing in healthy cats suggest that detecting FCV antibodies cannot replace routine vaccination against FCV [10]. A study by Spiri et al. informs the optimization of environmental control measures for FCV, based on an investigation of virus viability after a recent outbreak [11].

### 1.4. Herpesviruses

Zhang et al. investigated miRNAs in innate host-resistance to the common alphaherpesvirus, feline herpesvirus 1 (FHV-1) [12]. Their results suggest that miR-26a could suppress FHV-1 replication by enhancing type I IFN-induced antiviral signaling via host suppressor of cytokine signaling, SOCS5.

### 1.5. Retroviruses

Research on the pathogenic feline retroviruses, feline leukemia virus (FeLV) and feline immunodeficiency virus (FIV) is well covered in this Special Issue, with articles on epidemiology, pathogenesis, innate immunity, viral assembly and immunomodulators. FeLV was originally isolated from households of pedigree cats but increased awareness, improved diagnostics and effective control measures, including vaccination, have been accompanied by a changing epidemiology. An update on the prevalence of FeLV viremia of 2.3% among cats visiting veterinarians across 32 European countries is reported by the European Advisory Board on Cat Diseases [13]. Significant differences in FeLV prevalence between different countries was identified. Originating from southern Europe, being non-pedigree and access to outdoors were important predictors of FeLV status. In Australia, a study by Westman et al. on outcomes in FeLV outbreaks highlights the need to maintain control measures for FeLV even where the prevalence of antigenemia in the general population is low [14].

Antiviral antibody testing is the diagnostic screening test of choice for FIV infection. Frankenfeld et al. investigated a perceived increase in negative FIV ELISA results compared with Western blot [15]. They demonstrate that the current diagnostic sensitivity and specificity of FIV antibody ELISA (82% and 91%, respectively) in Europe are lower than in 1995 (98% and 97%). The authors suggest that this new challenge to FIV diagnosis could result from increased movement of pets and the introduction of new FIV isolates not recognized by screening assays.

Restriction factor APOBEC3 (A3) plays an important role in innate immunity to lentiviruses and is well studied in HIV infection. In FIV infection, A3 degradation is mediated by FIV viral infectivity factor (Vif), but the link between A3–Vif interactions and the biological activity of A3 requires clarification. Troyer et al. compared expression of A3 genes, A3Z2, A3Z3 and A3Z2-Z3, in cats infected with domestic cat FIV or puma FIV [16]. Overall, the authors conclude that the capacity of Vif to counteract A3 has a greater influence on lentiviral replication competence in cats than modulation of A3 expression.

Simoes et al. report that depletion of regulatory T cells did not improve innate immunity against *Listeria monocytogenes* in FIV-infected cats, and they suggest dendritic cell dysfunction as a factor [17]. Relevant to the establishment of lentiviral reservoirs, Asadian and Bienzle investigated the in vitro activity of interferons on antiviral lymphocyte protein SAMHD1, reporting that SAMH1 is inducible by IFN*γ*, and that overall activity is cell type- and compartment-specific [18]. Ovejero et al. used mutagenesis to study the role of Y176/L177 in FIV assembly and viral infectivity, demonstrating functional equivalents to its HIV-1 counterpart [19].

In a prospective clinical study, Gomez-Lucia et al. evaluated low-dose oral recombinant human interferon-*α* (rHuIFN-*α*) as an immunomodulatory treatment in FeLV- or FIV-infected cats, reporting clinical improvements during treatment [20]. However, a rebound in viral parameters after cessation of treatment points to the need to clarify the true efficacy and potential adverse effects of rHuIFN-*α* treatment on FIV progression.

Use of prednisolone and cyclosporine A (CsA) is sometimes indicated in FIV-infected cats to treat secondary diseases, such as immune-mediated disease, stomatitis, or lymphoma, but the effects of these drugs on FIV replication and persistence are poorly understood. Miller et al. investigated the immunomodulatory effects of prednisolone and CsA in cats with FIV infection, reporting acute, transient increases in FIV DNA and RNA and CD4+ lymphocytes, as well as cytokine expression, which favored a shift toward a Th2 response [21].

### 1.6. Novel Viruses

In cats, as in other species, novel viruses are being discovered much faster than their biological relevance can be ascertained. An in-depth review from DiMartino et al. tackles the gamut of novel enteric viruses including noroviruses, kobuviruses, and novel parvoviruses, updating on the etiology, epidemiologic, pathogenetic, clinical, and diagnostic aspects of these viral infections in cats [22].

Understanding the cause(s) of chronic kidney disease (CKD), a major cause of death in pet cats, is a holy grail in feline medicine. The discovery of a feline morbillivirus (FeMV) in Hong Kong a decade ago caused much excitement because a link with CKD was postulated [23]. Many studies since have addressed a potential association between FeMV infection and CKD, but a clear picture has yet to emerge. A timely review by Choi et al. brings the current state of knowledge of FeMV into perspective [24].

The feline hepatitis B virus (HBV) homologue, domestic cat hepadnavirus (DCH), was discovered in Australia in 2018 [25]. Using in situ hybridization (ISH) and PCR in archived feline liver biopsies, Pesavento et al. report a compelling association between DCH and chronic hepatitis and hepatocellular carcinoma in cats that mirrors HBV-associated hepatopathies [26]. Whether DCH is a feline pathogen remains to be seen.

New data on gammaherpesviruses come from Hendrikse et al., who identified a novel gammaherpesvirus in Canada lynx (*Lynx canadensis*), LcaGHV1, in 36% of samples from USA and 17% from Canada [27]. Novacco et al. report a molecular prevalence of FcaGHV1, the domestic cat gammaherpesvirus, of 5.5% to 6.0% among domestic cats in Switzerland. Although the potential for FcaGHV1 to be oncogenic in a minority of cases has been considered, the authors found that 0/17 cat tissues from cats with lymphoma tested positive for FcaGHV1, highlighting the need for the pathogenic potential of FcaGHV1 to be further evaluated [28].

Papillomaviruses infecting humans have been suggested to play a role in cutaneous squamous cell carcinoma (SCC) [29]. A novel feline papillomavirus, tentatively assigned FcaPV6, was reported by Carrai et al. in association with feline SCC [30]. Classification of FcaPV6 in a new genus alongside FcaPVs 3, 4 and 5 is supported.

## 2. Concluding Remarks

This Special Issue collates important advances in basic and applied research in feline viruses and viral diseases and highlights some of the many questions still to be answered as the field expands and adapts to meet the world’s needs.
